# Karyotypical Confirmation of Natural Hybridization between Two Manatee Species, *Trichechus manatus* and *Trichechus inunguis*

**DOI:** 10.3390/life12050616

**Published:** 2022-04-20

**Authors:** Edivaldo H. C. de Oliveira, Anderson J. B. Gomes, Alexandra F. Costa, Renata Emin-Lima, Cibele R. Bonvicino, Maria C. Viana, Laura M. A. Reis, Marcelo D. Vidal, Mirella V. G. Cavalcanti, Fernanda L. N. Attademo, Fábia O. Luna, Salvatore Siciliano

**Affiliations:** 1 ICEN, Universidade Federal do Pará, Belém 66075-110, PA, Brazil; 2 SEAMB, Instituto Evandro Chagas, Ananindeua 67030-000, PA, Brazil; 3 Instituto Federal do Pará, Abaetetuba 68440-000, PA, Brazil; anderson.gomes.ifpa@gmail.com; 4 Museu Paraense Emílio Goeldi, Belém 66077-830, PA, Brazil; alexandrafernandescosta@gmail.com (A.F.C.); sotalias@gmail.com (R.E.-L.); 5 Instituto Bicho D’água, Belém 66623-311, PA, Brazil; 6 Instituto Nacional do Câncer (INCA), Rio de Janeiro 20231-050, RJ, Brazil; cibele.bonvicino@gmail.com (C.R.B.); mcarolviana@gmail.com (M.C.V.); 7 Departamento de Biologia Animal, Instituto de Biologia, Universidade Estadual de Campinas (UNICAMP), Campinas 13083-862, SP, Brazil; 8 Núcleo de Gestão Integrada (ICMBio), São Luís 65025-470, MA, Brazil; laura.reis@icmbio.gov.br; 9 Centro Nacional de Pesquisa e Conservação da Sociobiodiversidade Associada a Povos e Comunidades Tradicionais (CNPT/ICMBio), São Luís 65025-470, MA, Brazil; marcelo.derzi.vidal@gmail.com; 10 Instituto Brasileiro do Meio Ambiente e dos Recursos Naturais Renováveis (IBAMA/CETAS), Macapá 68909-329, AP, Brazil; mirella.cavalcanti@ibama.gov.br; 11 Centro Nacional de Pesquisa e Conservação de Mamíferos Aquáticos (ICMBio/CMA), Instituto Chico Mendes de Conservação de Biodiversidade, Santos 11050-031, SP, Brazil; niemeyerattademo@yahoo.com.br (F.L.N.A.); fabialunacma@gmail.com (F.O.L.); 12 Departamento de Ciências Biológicas, Escola Nacional de Saúde Pública/Fiocruz, Rio de Janeiro 21041-210, RJ, Brazil

**Keywords:** introgression, karyotypes, hybridization, heterozygosity, sympatry, fertility

## Abstract

Two species of manatees are found in Northern Brazil—the Antillean manatee (*Trichechus manatus*), which is found along the coast from Florida to Northeastern Brazil, and the Amazonian manatee (*Trichechus inunguis*), endemic to the Amazon drainage basin. These species show a sympatric distribution in the region of the Marajó Archipelago, an estuarine area surrounding the Amazon River mouth. There is evidence of the occurrence of interspecific hybrids in this area, based on mitochondrial DNA analyses, although the use of nuclear markers has not corroborated this proposal. Considering that these species show very distinct karyotypes, despite being closely related (2*n* = 48 in *T. manatus* and 2*n* = 56 in *T. inunguis*), hybrids would present distinct chromosome numbers. Based on this, we conducted cytogenetic analyses using classic and molecular techniques in three calves found stranded in the Marajó Island and Amapá coast. The results showed that one of them, morphologically classified as *T. inunguis*, presented the correspondent karyotype, with 2*n* = 56. However, the other two, which were phenotypically similar to *T. manatus*, showed 2*n* = 49. Despite the same diploid number, their G-banding patterns revealed some differences. The results of the distribution of some microsatellite sequences have also confirmed the heterozygosity of some chromosomal pairs in these two individuals. These results are the first indubitable confirmation of the occurrence of natural hybrids between *T. manatus* and *T. inunguis*, and also brings about some issues concerning the viability of hybrids, considering that these two individuals do not correspond to an F1 hybrid, but instead, both presented a possible F2 karyotype.

## 1. Introduction

The aquatic herbivorous mammals known as manatees belong to genus *Trichechus* (Sirenia, Trichechidae), with three extant species, two of which are found in Brazil: the Antillean manatee (*T. manatus*) and the Amazonian manatee (*T. inunguis*). While *T. manatus* is found in coastal waters from Florida to Northeastern Brazil, *T. inunguis* is restricted to freshwater, and is found in rivers and lakes in the Amazon River basin [1,2]. 

However, despite this, these two species are found in sympatry in the estuarine region of the Marajó Archipelago, a complex area near the Amazon delta, with rivers, lakes and where fresh and saltwater mix in distinctive patterns according to the rainfall regime [3,4,5,6]. Because of this, the possible occurrence of hybridization between *T. manatus* and *T. inunguis* has been analyzed by means of molecular studies, based on both mitochondrial and molecular DNA markers [1,7,8]. Unfortunately, the results of these studies are currently inconclusive [9]. Therefore, while mitochondrial DNA (mtDNA) analyses have indicated the occurrence of interspecific hybrids in the estuarine sympatric region [1,7], the sequencing of nuclear (nuDNA) markers did not find evidence of hybridization in individuals sampled in the area, including individuals from the Guianas, proposed as part of the hybrid zone [8]. 

Cytogenetics is a primary tool that could be used to identify hybrid individuals between species with different karyotypes, in which chromosomes of the parental species can be easily differentiated by classical techniques, such as G-banding [10]. Concerning manatees, karyotypical studies are still limited, and most of the analyses were based on classical cytogenetics techniques [11,12,13,14,15,16]. The results so far have shown that South American manatees have different chromosome complements, with 2*n* = 48 in *T. manatus* [12,14,16] and 2*n* = 56 in *T. inunguis* [7,11,13]. In addition, heterochromatic blocks are restricted to the centromeric region both in *T. manatus* and *T. inunguis*, which also showed similar results in relation to nucleolar organizer regions, observed by Ag-NOR technique in only one chromosome pair [12,13]. Considering the number of chromosomal arms, the different karyotypes observed between these two species could only be explained by the occurrence of both intrachromosomal and interchromosomal rearrangements, such as inversions, translocations and fusions, although the real process will only be elucidated after applying comparative chromosome painting to confirm homeology among chromosome pairs [13,14]. Unfortunately, up to now, ZOO-FISH is restricted to *T. manatus* [17,18], and the results strongly supported the close relationship between sirenians and elephants (Tethytheria) supporting the clade Paenungulata, which includes Sirenia, Proboscidea, and Hyracoidea [18]. Interestingly, although these two species show clear chromosomal differences [11,12,13,14,15,16,17,18], there are no karyological studies in individuals from the sympatric area, except for one male found in illegal captivity, which was confirmed as being a hybrid between *T. manatus* and *T. inunguis* by mitochondrial and nuclear DNA data, as well as cytogenetic analysis [1,8]. 

Based on the fact that the chromosomal differences observed between *T. manatus* and *T. inunguis* could be used to reveal unequivocal evidence of the occurrence of hybridization, we analyzed the karyotype of three calves found stranded on beaches along the Marajó Archipelago and Amapá coast, by means of classical and molecular cytogenetics. The results raised important issues not only regarding natural hybridization, but also possible fertility of hybrids, despite their chromosomal heterozygosity.

## 2. Materials and Methods

### 2.1. Samples and Chromosome Preparation

**Figure 1:**
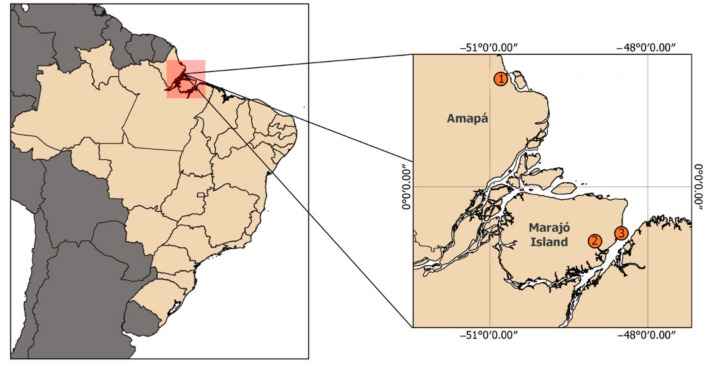
Geographical location of the sites in Brazil where individuals analyzed in this study were either rescued, rehabilitated or both. Based on morphological features, individuals 1 and 3 were phenotypically classified as *Trichechus manatus*, and individual 2 as *Trichechus inunguis*.

**Figure 2:**
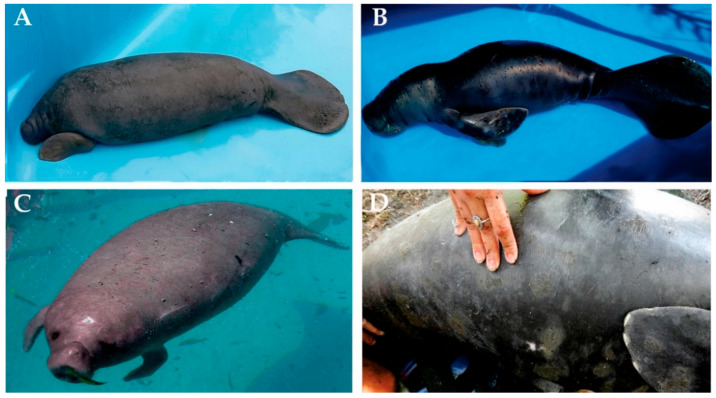
Morphological features of *Trichechus* specimens examined in this study: (**A**) Individual from locality 1, a typical *T. manatus* in general appearance, with greyish color and presence of nails; (**B**) individual from locality 2, a typical *T. inunguis* in general appearance, with blackish coloration and absence of nails; and (**C**,**D**) individual from locality 3, coloration that resembles an Amazonian manatee (*T. inunguis*), but has nails as an Antillean manatee (*T. manatus*). In addition, its body size also follows the general description of *T. inunguis*. Photos by: (**A**) Fernanda Attademo, (**B**–**D**) Instituto Bicho D’água files.

Blood samples were collected in heparinized sterile syringes, from three young males of *Trichechus* sp. They were rescued from an area including the Amapá State and the Marajó Archipelago (Figure 1). Morphological traits of these individuals are described in Figure 2. Samples were immediately placed on ice and transported to the laboratory for cell culture procedures. Whole blood culture followed the protocol according to Moorhead et al. [19], with modifications. Briefly, ten drops of whole blood were added in 5 mL of RPMI 1640 (SIGMA ALDRICH, St Louis, MO, USA) enriched with 10% fetal calf serum (GIBCO/Thermo Fisher Scientific, Santa Clara, CA, USA) and 2% phytohemagglutinin (GIBCO/Thermo Fisher Scientific, Santa Clara, CA, USA) and incubated for 72 h at 37 °C. For metaphase arrest, 100 µL KaryoMAX Colcemid solution (GIBCO/Thermo Fisher Scientific, Santa Clara, CA, USA) were added 1 h before the 72nd hour. Next, the material was centrifuged at 1000 rpm for 10 min, and after discarding the supernatant, 5 mL of KCl (0.075 M) was added, and the material was incubated for 20 min at 37 °C. Afterwards, we proceeded with fixation and washing with fixative solution (3 methanol:1 acetic acid). Chromosome suspensions were dropped on slides and submitted to different classical and molecular cytogenetic techniques.

### 2.2. Classical Cytogenetic Techniques

For the definition of diploid number and chromosome morphology, an average of 20 metaphases in conventional staining (5% Giemsa in phosphate buffer pH 6.8) were analyzed for each individual. We followed the chromosome nomenclature as previously published [13,14]. The distribution of constitutive heterochromatic blocks was analyzed by C-banding [20], and the homology of chromosome pairs was defined using G-banding [21].

### 2.3. Fluorescent In Situ Hybridization (FISH)

Different repetitive DNA sequences were used as probes in the fluorescent in situ hybridization (FISH) experiments. Biotin-labeled 18/28SrDNA probes were used to map the clusters of rDNA [22]. Telomeric probes were generated by PCR using primers (TTAGGG)_5_ and (CCCTAA)_5_ without DNA template [23] and labeled with digoxigenin-11-dUTP using Nick Translation Mix (ROCHE, Belgium, Switzerland), according to the manufacturer’s protocol. FISH experiments with rDNA and telomeric probes followed Yang et al. [24].

Five microsatellite probes (short repeats) were used: (TA)_15_, (CGG)_10_, (GC)_15_, (GAG)_10_ and (CAG)_10_. Probes were directly labeled with Cy3 during synthesis (EXXTAND BIOTECNOLOGIA, São Paulo, Brazil) and we followed the hybridization procedures described by Kubat et al. [25]. 

### 2.4. Microscopic Analyses and Image Capture and Processing

Slides submitted to classical cytogenetic procedures (conventional, G- and C-banding) were analyzed using a Leica DM-100 optical microscope. Camera control and image acquisition was performed using GenAsis software (Applied Spectral Imaging, Carlsbad, CA, USA). FISH experiments were analyzed using a Zeiss Imager II microscope (ZEISS, Jena, Germany), and images were captured and processed using a CCD camera and Axiovision 4.8 software (ZEISS, Jena, Germany).

## 3. Results

### 3.1. Karyotype Description: Diploid Number, G-Banding Patterns and Distribution of Constitutive Heterochromatin Blocks

**Figure 3:**
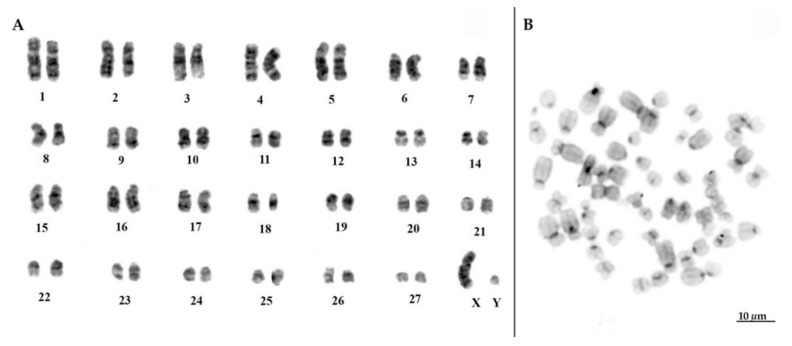
(**A**) G-banded karyotype of *Trichechus inunguis* (individual 2) with typical 2*n* = 56 chromosomes; (**B**) C-banded metaphase show a low amount of heterochromatin, restricted to the centromeric regions of the chromosomes.

One of the individuals classified as *T. inunguis* showed 2*n* = 56, with chromosome morphology, G- and C- banding patterns similar to previous descriptions of the karyotype of this species [8,13,14]. Hence, autosomes comprised 15 biarmed pairs and 12 acro/telocentric pairs; the X chromosome was submetacentric and the Y acrocentric (Figure 3A). Concerning the distribution of constitutive heterochromatin, they were restricted to the pericentromeric region (Figure 3B).

**Figure 4:**
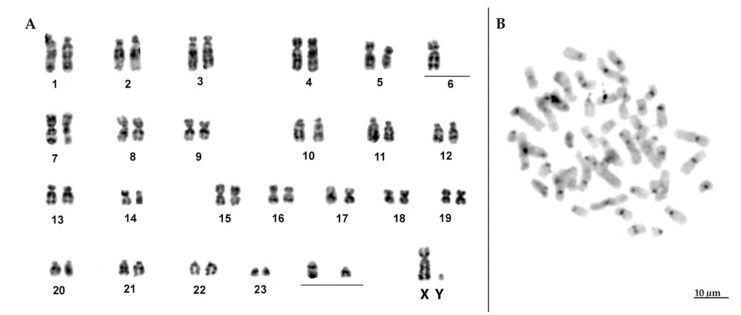
(**A**) G-banded karyotype of individual 1 with 2*n* = 49. Dash indicates chromosome 6, homologous to two unpaired acrocentric chromosomes; (**B**) C-banded metaphase showing the low amount of heterochromatin in the centromeric region of the chromosomes.

**Figure 5:**
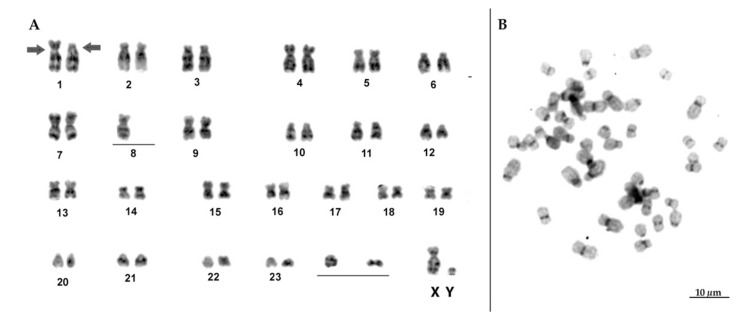
(**A**) G-banded karyotype of individual 3 with 2*n* = 49 chromosomes. Arrows show the difference in the centromeres position on chromosome 1; dash shows chromosome 8, homologous to two unpaired acrocentric chromosomes; (**B**) C-banded metaphase indicating the heterochromatin in the centromeric region of the chromosomes.

The other two young males (1 and 3 in the map), phenotypically similar to *T. manatus*, showed a diploid number with 49 chromosomes, diverging from the typical diploid number for *T. manatus* (2*n* = 48). Interestingly, despite having the same fundamental number, with 37 biarmed and 10 one-armed chromosomes, the G-banding patterns revealed some slight differences between their chromosome complements. Hence, while one individual showed pair 6 formed by heterozygotic elements—one submetacentric element homologous to two acrocentric chromosomes (Figure 4A)—the second individual with 2*n* = 49 showed pair 8 formed by three elements (Figure 5A). In relation to the distribution of heterochromatic blocks, both karyotypes showed heterochromatin restricted to the centromeric regions of the chromosomes (Figure 4B and Figure 5B).

### 3.2. Distribution of Repetitive Sequences: 18/28rDNA, Telomeric Sequences and Microsatellites

**Figure 6:**
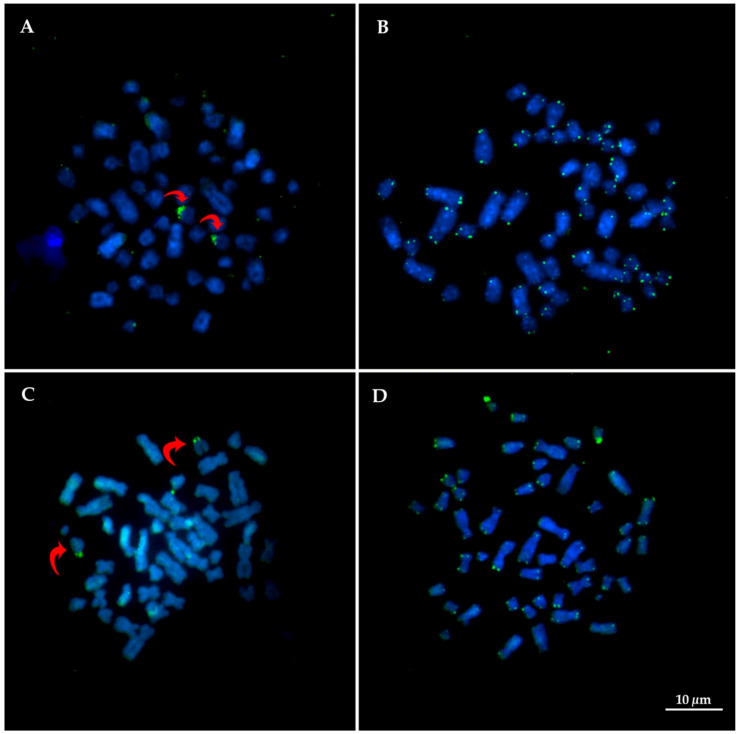
FISH experiment using 18/28S rDNA and telomeric sequence in metaphases of *T. inunguis* (**A**,**B**) and a hybrid individual (**C**,**D**), respectively. Red arrows show NOR site.

Results from hybridization using 18/28r DNA probes have confirmed only one NOR-bearer pair, corresponding to pair 20 in both species (Figure 6A,C). Telomeric sequence was observed only in the end of chromosome arms, without interstitial telomeric site (Figure 6B,D).

**Figure 7:**
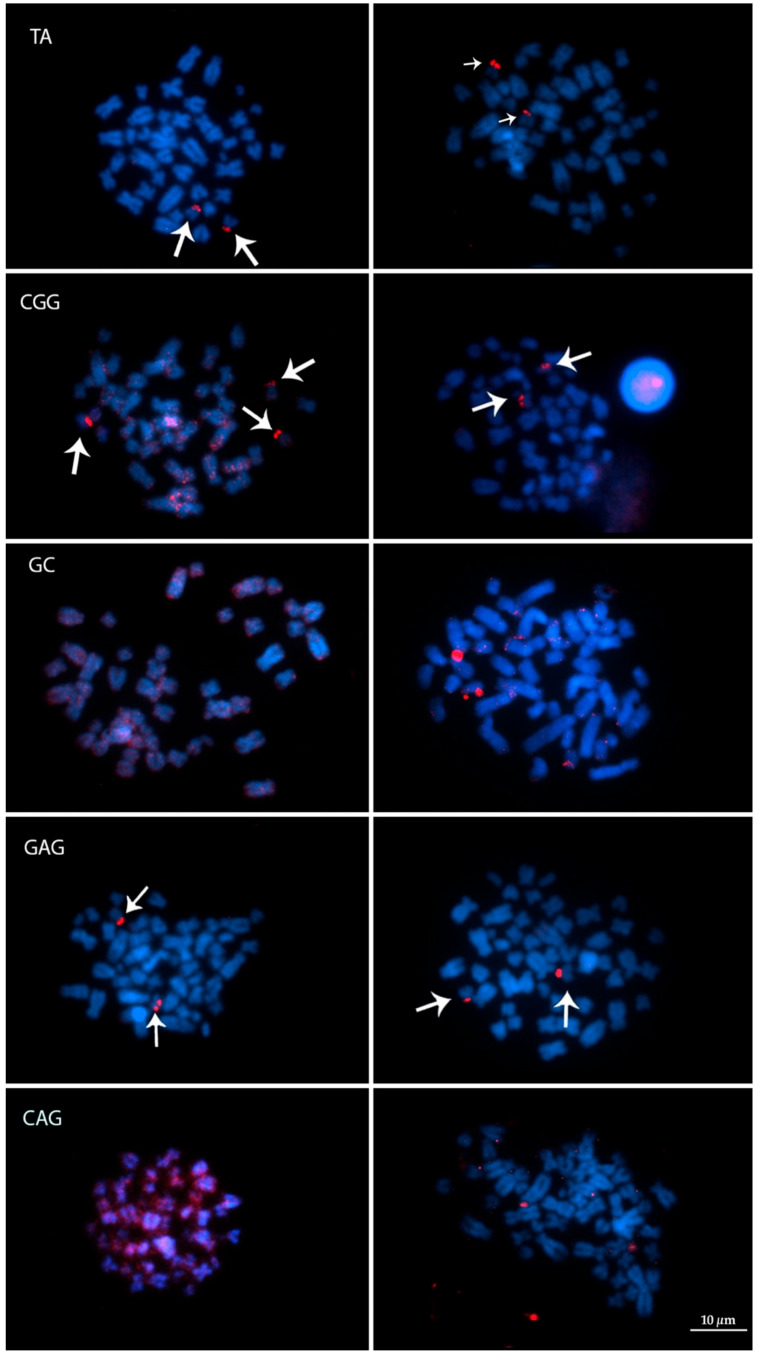
FISH experiment using microsatellite sequences (TA)_15_, (CGG)_10_, (GC)_15_, (GAG)_10_ and (CAG)_10_ in both hybrid specimens. Metaphases on the left correspond to individual 1, and on the right to individual 3. Arrows indicate the clusters of theses sequences in the chromosomes.

Due to the low number of metaphases obtained in individual 2 (*T. inunguis*), the experiments using five microsatellite repeats were mapped only in the karyotypes of the two hybrid specimens. The sequences (TA)_15_, (GAG)_10_ and (CGG)_10_ produced signals in clusters colocalized with the NOR (pair 20). However, (CGG)_10_ produced an additional signal in one of the chromosomes of pair 14 in individual 2. The microsatellite (CAG)_10_ showed dispersive patterns in the genome for both specimens and the sequence (GC)_15_ also had a dispersive pattern, but with tenuous signals in the centromere and telomeres for both specimens. Representative figures for each microsatellite are summarized in Figure 7.

## 4. Discussion

The karyotype can be considered a global map of the nuclear genome of a species [26]. Therefore, descriptions of chromosome sets have been essential for drawing conclusions concerning not only taxonomic revision in some groups of species, but also in the identification of interspecific hybrids, especially between species with very distinct karyotypes, when hybrid karyotypes are clearly evidenced [27,28]. Curiously, in spite of the clearly different karyotypes found in *T. manatus* and *T. inunguis*, cytogenetic analyses of individuals with confirmed origins from natural populations had not been explored as an alternative to clarify the occurrence of natural hybridization between them. One of the individuals analyzed herein was phenotypically classified as *T. inunguis*, and its karyotype also confirmed this classification. This specimen was rescued inland of the Marajó Island, in a freshwater environment (Cachoeira do Arari, PA), and showed a karyotype similar to previous reports for *T. inunguis*, with 2*n* = 56, and the same chromosomal morphology [11,13]. However, the other two animals, found closer to the coastline, showed a new diploid number for genus *Trichechus,* with 2*n* = 49, and the G-banding analyses revealed unequivocal evidence that they had heterozygote chromosome pairs, confirming them as hybrids. In this sense, our study is the first report of natural hybrids between *T. manatus* and *T. inunguis* based on chromosomal evidence. 

The first invocations of a possible hybrid zone between the two species of manatees found in South America were based on results from mtDNA control region sequences [29], although the authors described human-mediated transplantation as a possible and simpler explanation to their findings, following the information from the zoo records. Subsequent studies using mtDNA markers have also found evidence for the occurrence of hybridization between *T. manatus* and *T. inunguis*, suggesting the existence of a hybrid zone and a hybrid swarm along the coastline of the Guianas [7]. Interestingly, the occurrence of hybrids in this area has not been supported by the results from the analyses of nuDNA markers, which have not found any indication of hybrids, except for one individual, which was found in captivity and also showed chromosomal evidence of being a hybrid [1,8].

This animal, a male, showing a karyotype with 2*n* = 50, was rescued from captive conditions in Oiapoque (AP, Brazil), a locality within the supposed hybrid zone, and is the only report in literature of a diploid number different to the ones observed in *T. manatus* and *T. inunguis*. Considering that an F1 hybrid between *T. manatus* and *T. inunguis* would have 2*n* = 52, it was suggested that this karyotype would correspond to an F2 backcross between an F1 hybrid female, with 2*n* = 52, and a male *T. manatus* [1]. In addition to the chromosomal evidence, this hybrid individual, which morphologically resembled *T. manatus*, showed molecular evidence of hybridization, both by mtDNA and nuDNA markers [7,8]. Therefore, although chromosomal differences have the potential to be a primer post-zygotic barrier, promoting reproductive isolation by producing hybrids with infertility or reduced fertility [30,31], this does not seem to be the case of manatees.

While previous research has focused mainly on molecular markers, our results provided a new insight to the occurrence of natural hybrids between these manatees based on karyological studies. Firstly, since the two hybrid individuals analyzed herein have 49 chromosomes, their diploid numbers do not match the result of an F1 between *T. manatus* and *T. inunguis* as well, showing different combinations of chromosomes, and supporting the potential fertility of interspecific hybrids. Even more interesting, although both individuals have 2*n* = 49, the G-banding patterns suggest that they exhibit different combinations of the parental karyotypes. Hence, one of them showed pair 8 formed by a biarmed element from *T. manatus* paired with the two acrocentric homeologous, from *T. inunguis* (Figure 5A). In contrast, the other hybrid had pair 8 formed by two submetacentric elements, from *T. manatus*, while in pair 6 we could identify one submetacentric chromosome from *T. manatus*, paired with the two acrocentric homologous chromosomes from *T. inunguis* (Figure 4A). In any case, both hybrids would carry at least one trivalent each. Moreover, other pairs had the same morphology and banding pattern in both species and could not be identified as heterozygotes by classical cytogenetics. In addition, the slight differences observe in the distribution of microsatellite sequences, an extra signal produced by (CGG)_10_ in one of the hybrids, may indicate that it corresponds to a chromosome pair in which each homologous came from one of the two different species. Unfortunately, we could not analyze the distribution of these sequences in the *T. inunguis* species due the low number of metaphases obtained. The distribution of microsatellite sequences in the karyotype of manatees is still poorly studied, with only one previous report showing the characterization and distribution of one satellite sequence in *T. manatus* and *T. inunguis* [32]. Nevertheless, our results indicate that microsatellites could represent useful markers for the detection of heterozygous chromosome pairs.

Secondly, the description of hybrid individuals not corresponding to F1 hybrids builds on evidence of fertile hybrids. The role of chromosomal rearrangements as reproductive barriers and possible role in speciation has been extensively debated, being usually associated to hybrid lower fitness, especially between species with either different chromosome number, morphology or both [33]. In addition, some authors argue that hybridization is less common in mammals than in other vertebrates, not only based on meiotic disruption, due to chromosomal rearrangements, but also on account of other mechanisms, such as genomic imprinting [30,31,33,34]. Accordingly, chromosomal rearrangements could also suppress recombination, protecting the genome from introgression [35]. In addition, we must consider that different degrees of fertility may exist, including cases of male-restricted infertility [30,31,33]. The extremely small number of analyzed hybrids leaves these issues open, but our findings are indicative of fertility, or at least partial reproductive viability, of F1 individuals.

Apart from chromosome studies, an issue that needs to be addressed is the discordance between results from mtDNA and nuDNA obtained in studies sampling individuals from the supposed hybrid zone [8]. In this sense, there are some reports showing that concordant patterns are not always the results obtained when using mtDNA and nuDNA [36,37]. In addition, mtDNA introgresses more easily than nuDNA [37,38,39]. Hence, although not detected by the nuDNA markers, gene flow between the two species of manatees may have caused or at least contributed to these conflicting results. As an example, a study involving two species from genus *Rhinella* (Amphibia, Anura) from Brazil, showed highly divergent nuDNA, while mtDNA was very similar, and this unidirectional introgression was explained by the occurrence of interspecific hybridization [36]. 

Concerning interspecific hybridization, it has been documented in a lower proportion of species in mammals when compared to other groups of vertebrates, probably due to some intrinsic features of this class [30]. However, this number has been continually increasing with the use of modern molecular tools [30,37]. In order Cetacea, for example, there are records of hybrids in 20% of species within this group, and the F1 hybrid fertility may be facilitated by the prominent karyological uniformity observed in this order [40]. 

Still concerning the impact of chromosomal differences, we have to mention reports of some close related species pairs belonging to family Bovidae in which the comparison between mtDNA and nuDNA markers have revealed cases of introgression, when the genome of one species has been transferred to another species [41,42,43,44,45]. Besides being closely related species, natural hybridization could be facilitated by characteristics, such as sharing the same habitat and having karyotypes that allowed the production of viable gametes, at least in females [45]. Our evidence showed that these features are observed in these manatees, at least in the area where *T. manatus* and *T inunguis* are sympatric. In addition, the previously described hybrid, found in captive conditions, was supposed to be an F2 individual resulted from a hybrid female and a pure *T. manatus* male [1], confirming that at least female hybrids show some fertility. Following this line of interpretation, F1 hybrid females would be responsible for the introgression, while F1 hybrid males may be sterile (Haldane’s rule) or show reduced fitness, preventing paternal genes introgression. The occurrence of population bottleneck, caused by the drastic population reduction observed in both species of manatees, associated to the natural occurrence of hybrids would facilitate the process of introgression and fixation of the new genome characteristics by genetic drift [46].

This study has potential limitations. For instance, the identification of homologous chromosomes was based on G-banding, therefore being subject to mispairing, considering that the metaphases were not in an optimal stretching level. However, the differences observed in the diploid numbers and chromosomal morphology were sufficient to suggest that these karyotypes were derived from hybridization events. Therefore, in conclusion, our results unequivocally demonstrate the natural occurrence of hybrids between *T. manatus* and *T. inunguis* in the region surrounding the Amazon River mouth and the Marajó Archipelago, where these species are found in sympatry. In addition, our findings support the fact that hybrids are fertile, at least partially. The observation of hybrids, despite the very small size of the sampled individuals, raises many questions concerning the frequency and consequences of hybridization. Considering that hybridization is part of the evolutionary process, forthcoming studies are necessary to analyze the biology and stability of hybrid populations, and to determine the consequences of the already existent introgression in the parental species. 

## Data Availability

Not applicable.

## References

[1] Vianna J.A., Bonde R.K., Caballero S., Giraldo J.P., Lima R.P., Clark A., Marmontel M., Morales-Vela B., De Souza M.J., Parr L. (2006). Phylogeography, phylogeny and hybridization in Trichechid sirenians: Implications for manatee conservation. Mol. Ecol..

[2] Marsh H., O’Shea T.J., Reynolds III J.E. (2011). Ecology and Conservation of Sirenia: Dugongs and Manatees.

[3] Domning D.P. (1981). Distribution and status of manatees *Trichechus* spp. near the mouth of the Amazon River, Brazil. Biol. Conserv..

[4] Luna F.O., Lima R.P., Castro D.F., Vianna J.A. Capture and utilization of the Amazonian manatee *Trichechus inunguis* in the state of Amazonas, Brasil. Proceedings of the 14th Biennal Conference of the Biology of Marine Mammals.

[5] Luna F.O., Araújo J.P., Oliveira E.M., Hage L.M., Passavante J.Z.O. (2010). Distribuição do peixe-boi marinho, *Trichechus manatus manatus*, no litoral norte do Brasil. Arq. De Ciências Do Mar.

[6] Sousa M.E.M., Martins B.M.L., Fernandes M.E.B. (2013). Meeting the giants: The need for local ecological knowledge (LEK) as a tool for the participative management of manatees on Marajó Island, Brazilian Amazonian coast. Ocean Coast. Manag..

[7] Lima C.S., Magalhaes R.F., Marmontel M., Meirelles A.C., Carvalho V.L., Lavergne A., Thoisy B.D., Santos F.R. (2019). A hybrid swarm of manatees along the Guianas coastline, a peculiar environment under the influence of the Amazon River plume. An. Da Acad. Bras. De Ciências.

[8] Luna F.D.O., Beaver C.E., Nourisson C., Bonde R.K., Attademo F.L., Miranda A.V., Torres-Florez J.P., de Sousa G.P., Passavante J.Z., Hunter M.E. (2021). Genetic connectivity of the West Indian manatee in the southern range and limited evidence of hybridization with Amazonian manatees. Front. Mar Sci..

[9] De Sá A.L.A., Baker P.K.B., Breaux B., Oliveira J.M., de Macedo Klautau A.G.C., Legatzki K., de Oliveira Luna F., Attademo F.L.N., Hunter M.E., Criscitiello M.F. (2022). Novel insights on aquatic mammal MHC evolution: Evidence from manatee DQB diversity. Dev. Comp. Immunol..

[10] Bulatova N., Shchipanov N., Searle J. (2007). The Seliger Moscow hybrid zone between chromosome races of common shrews an initial description. Russ. J. Theriol..

[11] Loughman W., Frye F., Herald E. (1970). The chromosomes of a male manatee*—Trichechus inunguis*. Husb. Res..

[12] White J.R., Harknesst D.R., Isaackst R.E., Duffield D.A. (1976). Some studies on blood of the Florida manatee, *Trichechus manatus latirostris*. Comp. Biochem. Physiol..

[13] Assis M., Best R., Barros R., Yassuda Y. (1988). Cytogenetic study of *Trichechus inunguis* (Amazonian manatee). Rev. Bras. De Genética.

[14] Gray B.A., Zori R.T., Mcguire P.M., Bonde R.K. (2002). A first generation cytogenetic ideogram for the Florida manatee (*Trichechus manatus latirostris*) based on multiple chromosome banding techniques. Hereditas.

[15] Hunter M.E., Mignucci-Giannoni A.A., Tucker K.P., King T.L., Bonde R.K., Gray B.A., McGuire P.M. (2012). Puerto Rico and Florida manatees represent genetically distinct groups. Conserv. Genet..

[16] Barros H., Meirelles A., Luna F., Marmontel M., Cordeiro-Estrela P., Santos N., Astúa D. (2017). Cranial and chromosomal geographic variation in manatees (Mammalia: Sirenia: Trichechidae) with the description of the Antillean manatee karyotype in Brazil. J. Zool. Syst. Evol. Res..

[17] Kellogg M.E., Burkett S., Dennis T.R., Stone G., Gray B.A., McGuire P.M., Zori R.T., Stanyon R. (2007). Chromosome painting in the manatee supports Afrotheria and Paenungulata. BMC Evol. Biol..

[18] Pardini A.T., O’Brien P.C.M., Fu B., Bonde R.K., Elder F.F.B., Ferguson-Smith M.A., Yang F., Robinson T.J. (2007). Chromosome painting among Proboscidea, Hyracoidea and Sirenia: Support for Paenungulata (Afrotheria, Mammalia) but not Tethytheria. Proc. R. Soc. B Biol. Sci..

[19] Moorhead P., Nowell P., Mellman W., Battips D., Hungerford D. (1960). Chromosome preparations of leukocites cultured from human peripheral blood. Exp. Cell Res..

[20] Sumner A. (1972). A simple technique for demonstrating centromeric heterochromatin. Exptl. Cell Res..

[21] Seabright M. (1971). A rapid banding technique for human chromosomes. Lancet.

[22] Cioffi M., Martins C., Centofante L., Jacobina U., Bertollo L. (2009). Chromosomal variability among allopatric populations of erythrinidae fish *Hoplias malabaricus*: Mapping of three classes of repetitive DNAs. Cytogenet. Genome Res..

[23] Ljdo J., Wells R., Baldini A., Reeders S. (1991). Improved telomere detection using a telomere repeat probe (TTAGGG)n generated by PCR. Nucleic Acids Res..

[24] Yang E., Carter N.P., Shiu L., Ferguson-Smith M.A. (1995). A comparative study of karyotypes of muntjacs by chromosome painting. Chromosoma.

[25] Kubat Z., Hobza R., Vyskot B., Kejnovsky E. (2008). Microsatellite accumulation on the Y chromosome in *Silene latifolia*. Genome.

[26] Graphodatsky A., Trifonov V., Stanyon R. (2011). The genome diversity and karyotype evolution of mammals. Mol. Cytogenet..

[27] Bakloushinskaya I.Y., Matveevsky S.N., Romanenko S.A., Serdukova N.A., Kolomiets O.L., Spangenberg V.E., Lyapunova E.A., Graphodatsky A.S. (2012). A comparative analysis of the mole vole sibling species *Ellobius tancrei* and *E. talpinus* (Cricetidae, Rodentia) through chromosome painting and examination of synaptonemal complex structures in hybrids. Cytogenet. Genome Res..

[28] Peres W.A.M., Bertollo L.A.C., Buckup P.A., Blanco D.R., Kantek D.L.Z., Moreira-Filho O. (2012). Invasion, dispersion and hybridization of fish associated to river transposition: Karyotypic evidence in *Astyanax* “bimaculatus group” (Characiformes: Characidae). Rev. Fish Biol. Fish..

[29] Garcia-Rodriguez A., Bowen B., Domning D., Mignucci-Giannoni A., Marmontel M., Montoyoa-Ospina R., Morales-Vela B., Rudin M., Bonde R.K., McGuire P.M. (1998). Introduction Phylogeography of the West Indian manatee (*Trichechus manatus*): How many populations and how many taxa?. Mol. Ecol..

[30] Shurtliff Q.R. (2013). Mammalian hybrid zones: A review. Mammal Rev..

[31] Grize S.A., Wilwert E., Searle J.B., Lindholm A.K. (2019). Measurements of hybrid fertility and a test of mate preference for two house mouse races with massive chromosomal divergence. BMC Evol. Biol..

[32] Valeri M., Dias G., do Espírito Santo A., Moreira C., Yonenaga-Yassuda Y., Sommer I., Kuhn G., Svartman M. (2021). First description of a satellite DNA in Manatees’ centromeric regions. Front. Genet..

[33] Horn A., Basset P., Yannic G., Banaszek A., Borodin P.M., Bulatova N.S., Jadwiszczak K., Jones R.M., Polyakov A.V., Ratkiewicz M. (2012). Chromosomal rearrangements do not seem to affect the gene flow in hybrid zones between karyotypic races of the common shrew (*Sorex araneus*). Evolution.

[34] Vrana P.B. (2007). Genomic imprinting as a mechanism of reproductive isolation in mammals. J. Mammal..

[35] Rieseberg L. (2001). Chromosomal rearrangements andspeciation. TRENDS Ecol. Evol..

[36] Sequeira F., Sodré D., Ferrand N., Bernardi J.A., Sampaio I., Schneider H., Vallinoto M. (2011). Hybridization and Massive mtDNA Unidirectional Introgression between the Closely Related Neotropical Toads *Rhinella marina* and *R. schneideri* Inferred from mtDNA and Nuclear Markers. http://www.biomedcentral.com/1471-2148/11/264.

[37] Mallet J. (2005). Hybridization as an invasion of the genome. Trends Ecol. Evol..

[38] Toews D.P.L., Brelsford A. (2012). The biogeography of mitochondrial and nuclear discordance in animals. Mol. Ecol..

[39] Vargas-Ramírez M., Carr J.L., Fritz U. (2013). Complex phylogeography in *Rhinoclemmys melanosterna*: Conflicting mitochondrial and nuclear evidence suggests past hybridization (Testudines: Geoemydidae). Zootaxa.

[40] Crossman C.A., Taylor E.B., Barrett-Lennard L.G. (2016). Hybridization in the Cetacea: Widespread occurrence and associated morphological, behavioral, and ecological factors. Ecol. Evol..

[41] Verkaar E.L.C., Nijman I.J., Beeke M., Hanekamp E., Lenstra J.A. (2004). Maternal and paternal lineages in cross-breeding bovine species. Has wisent a hybrid origin?. Mol. Biol. Evol..

[42] Ropiquet A., Hassanin A. (2005). Molecular phylogeny of caprines (Bovidae, Antilopinae): The question of their origin and diversification during the Miocene. J. Zool. Syst. Evol. Res..

[43] Willows-Munro S., Robinson T.J., Matthee C.A. (2005). Utility of nuclear DNA intron markers at lower taxonomic levels: Phylogenetic resolution among nine *Tragelaphus* spp.. Mol. Phylogenetics Evol..

[44] Hassanin A., Ropiquet A., Couloux A., Cruaud C. (2009). Evolution of the mitochondrial genome in mammals living at high altitude: New insights from a study of the tribe Caprini (Bovidae, Antilopinae). J. Mol. Evol..

[45] Hassanin A., An J., Ropiquet A., Nguyen T.T., Couloux A. (2013). Combining multiple autosomal introns for studying shallow phylogeny and taxonomy of *Laurasiatherian mammals*: Application to the tribe Bovini (Cetartiodactyla, Bovidae). Mol. Phylogenetics Evol..

[46] Hassanin A., Houck M.L., Tshikung D., Kadjo B., Davis H., Ropiquet A. (2018). Multi-locus phylogeny of the tribe Tragelaphini (Mammalia, Bovidae) and species delimitation in bushbuck: Evidence for chromosomal speciation mediated by interspecific hybridization. Mol. Phylogenetics Evol..

